# Adolescent Loneliness When a Parent Has Cancer: A Qualitative Systematic Review

**DOI:** 10.1002/pon.70148

**Published:** 2025-04-08

**Authors:** Lydia Mckeown, Kerry Campbell, Martin Dempster, Jenny Groarke, Lisa Graham‐Wisener

**Affiliations:** ^1^ School of Psychology Centre for Improving Health‐Related Quality of Life Queen's University Belfast Belfast UK; ^2^ School of Psychology National University of Ireland Galway Ireland

**Keywords:** adolescence, cancer, loneliness, mental health, parental cancer, psycho‐oncology, psychosocial, qualitative, social isolation, youth

## Abstract

**Objectives:**

The aim of this systematic review was to synthesise the available qualitative evidence relating to adolescent loneliness (aged 10–19) when a parent has cancer. This included considering how adolescents experience loneliness when their parent has cancer and exploring the perceived factors that influence this experience.

**Methods:**

A qualitative systematic literature review was conducted to synthesise and critically evaluate loneliness in adolescents experiencing parental cancer. The JBI qualitative systematic review approach was followed throughout, including quality appraisal, data extraction and a meta‐aggregative synthesis. The reports were screened by two independent reviewers and conflicts were resolved by a third reviewer. The review was pre‐registered on PROSPERO (CRD42023409596).

**Results:**

A total of 17 papers were included, published between 2007–2024. Findings indicate that young people experiencing parental cancer are lonely and deal with overwhelming emotions alone. Loneliness is intensified by a lack of understanding from peers and family constrained communication. Family and social support may protect young people against experiencing loneliness.

**Conclusions:**

Although there is a dearth of research on parental cancer which has focused on loneliness in this population, the analysis revealed that experiences of loneliness are indeed apparent in this cohort. Future research should focus on interviewing this population to develop a more comprehensive understanding of their lived experience.

## 1 Background

Cancer is a disease which affects the entire family unit. Between 14.7% and 24.7% of cancer patients have children under 18, meaning that parental cancer impacts a large amount of young people [1]. This is distressing for a child of any age, but particularly during adolescence [2, 3, 4]. Adolescence is an important developmental period which takes place aged 10–19 years old [5], where young people experience rapid physical maturation and changes within their social roles [6]. It is a time of increased vulnerability toward mental illness [7] including anxiety, depression, and suicidal behaviour [8, 9]. Research also indicates that adolescence is a time of vulnerability toward loneliness. Loneliness is a negative emotional experience resulting from the perception that an individual's social network is lacking in quality or quantity [10]. Systematic review data [11] reveals that loneliness is a significant issue among adolescents, with pooled prevalence rates ranging from 9.2% to 14.4%. Concerningly, adolescent loneliness is not only prevalent but increasing globally [12]. With a risk of mental health issues and loneliness commonly emerging during time in youth, young people experiencing the additional stress of parental cancer are placed in an especially vulnerable position. To effectively address loneliness across the world, it is essential to have a developed understanding of its main drivers [13]. However, loneliness is a subjective and unique lived experience [14] meaning that these drivers likely differ across various sub‐populations. With this understanding in mind, recent policy recommendations suggest that it is necessary to explore loneliness among specific subgroups [15] making this an opportune time to explore loneliness in young people experiencing parental cancer. It is important that researchers consider youth loneliness when a parent has cancer specifically as current conceptualisations of loneliness may not be applicable to adolescents who possibly experience loneliness differently than adults [16], especially when their parent is ill. Whilst we are aware that adolescents experiencing parental cancer are at risk of issues such as post‐traumatic stress disorder symptoms [17] disrupted functioning [18, 19], cancer‐related worry, stress, and decreased quality of life [20], much less is known about how they experience loneliness. A robust understanding of loneliness in young people experiencing parental caner is vital for future work to designs targeted interventions and optimise current services, with the view of improving the outcomes of young people when their parent has cancer.

A conceptual review of loneliness by Mansfield et al. [21] highlighted a qualitative study on loneliness during parental cancer by Karlsson et al. [22], which reports how young people feel deeply alone when their parent is ill. Despite this, no existing review has given dedicated focus to adolescent loneliness when a parent has cancer. There are several reviews on the impact of parental cancer on youth [3, 18, 19, 20, 23, 24, 25, 26, 27], however, only Walczak et al. [20] explicitly discusses ‘feelings of isolation’ in their findings. This suggests that loneliness in young people experiencing parental cancer have not been fully considered in the past. A qualitative review of existing evidence on adolescent's experiences of loneliness during parental cancer is imperative to develop a subjective and nuanced understanding of loneliness in this population, and to fully amplify the voices of young people navigating loneliness when their parent is ill. Increasing research and attention has been given to exploring cancer‐related loneliness in individuals diagnosed with cancer [28], survivors of cancer [29] and informal caregivers [30]. Despite this, there remains a dearth of knowledge regarding cancer‐related loneliness in dependent young people when their parent is ill. This is essential to address as parents with cancer have urged researchers to prioritise exploring psychological support and wellbeing of careers and families when someone has cancer, in the James Lind Alliance Priority Setting Partnership (PSP) exercise. The PSP also specifically highlighted a need to explore the psychological and social impacts on young people who have a parent (or parents) with cancer and how is best to support them.

A preliminary search of PROSPERO and Open Science Framework confirmed that there are no previous or current systematic reviews which aim to synthesize the existing literature pertaining to loneliness during adolescence when a parent has cancer. This review will examine qualitative peer‐reviewed publications relevant to adolescents experiencing parental cancer with the aim of extracting findings which relate to loneliness.

### 1.1 Aim

The aim of this systematic review was to synthesise the available qualitative evidence relating to adolescent loneliness aged 10–19 (World Health Organisation) when a parent has cancer. The research questions were:

1. How do adolescents experience loneliness when their parent has cancer?
2. What are the perceived factors that influence the experience of loneliness during parental cancer?

## 2 Method

### 2.1 Study Design

The review was conducted following the meta‐aggregative approach to synthesis according to the JBI methodology for qualitative systematic reviews [31]. The JBI meta‐aggregation approach involves extracting relevant quotations from each article into JBI software. Meta‐aggregation seeks to present the primary author's findings and does not aim to re‐interpret those findings. In the JBI software, quotations from every article are grouped based on similarity of meaning and then further aggregated into several synthesised findings. The inclusion and exclusion criteria can be seen in Table 1.This systematic review followed the reporting guidelines of the Preferred Reporting Items for Systematic Reviews and Meta‐Analyses Protocols (PRISMA‐P) statement [32]. A PRISMA 2020 checklist is included (Supporting Information S1). The systematic review was pre‐registered (PROSPERO; CRD42023409596) and can viewed at: https://www.crd.york.ac.uk/prospero/display_record.php?ID=CRD42023409596.

**TABLE 1:** Inclusion and exclusion criteria.

Inclusion criteria	Exclusion criteria
Population and settings	
Studies that included adolescents (aged 10–19) who had a parent diagnosed with any stage of cancer (curative or advanced), at any point during the young person's life Studies from all geographical regions	Studies not published in English (due to lack of translation services)
Outcomes and phenomena of interest	
Studies that include adolescents experiencing parental cancer, with the aim of extracting findings which related to loneliness Studies that include adolescents experiencing parental cancer with findings which discussed feeling ‘alone’ or ‘isolated’ and factors which influenced this experience such as a lack of support	Studies on parental cancer during adolescences that had no findings relating to loneliness Studies that did not include adolescents experiencing parental cancer with findings which discussed feeling ‘alone’ or ‘isolated’ and factors which influenced this experience such as a lack of support
Types of studies	
Qualitative designs and peer‐reviewed publications relevant to adolescents experiencing parental cancer were reviewed with findings relating to loneliness extracted Mixed method papers the qualitative information could be extracted Papers where at least half of the sample were aged between 10 and 19 years old In studies which looked at chronically ill parents, the paper was only included if at least half of the sample's parents had cancer	Intervention studies as these were not being evaluated Papers which focused *exclusively on life after the* death of a parent from cancer were excluded to ensure the review only included information pertaining to loneliness as opposed to grief Non‐empirical research (i.e., systematic reviews, editorials, opinion papers, case studies) was excluded, however relevant studies were harvested from them, where relevant

### 2.2 Search Strategy

An initial limited search of MEDLINE (Ovid) was undertaken to identify articles on the topic using the following initial keywords (Table 2).

**TABLE 2:** Search strategy terms.

Cancer OR malignan* OR neoplasms
**AND**
Offspring OR son or daughter or child
**AND**
Unmet needs OR distress OR impact OR bereavement OR bereave* OR coping

The text contained in the titles and abstracts of relevant articles, and the index terms were used to develop a full search strategy. The finalised search strategy (see Supporting Information S2) was adapted and applied to the following databases: MEDLINE (Ovid), CINHAL (EBSCO), PsycInfo (Ovid), EMBASE (Ovid). Searches were performed on 5th May 2023 and subsequently re‐performed on the 20th of July to include articles published since the initial search until present day. The time frame for the literature search was from 2007 to present‐day, to ensure the review captured young people's current experience of loneliness. Manual searches were also conducted by examining the reference lists of the included studies and identified reviews, and by performing citation searches on included articles.

### 2.3 Quality Assessment

Eligible studies were critically appraised for methodological quality by two independent reviewers (L.M. and K.C.) using the JBI Critical Appraisal Checklist Tools in JBI SUMARI [31]. Provided that a study met all the inclusion criteria, all studies, regardless of their methodological quality, underwent data extraction and synthesis. Any disagreements that arose between the reviewers were resolved with a third reviewer (M.D.).

### 2.4 Data Extraction

Extraction of descriptive qualitative data was completed by two independent reviewers (L.M. and K.C.), using the qualitative JBI data extraction tools in JBI SUMARI [33]. Specific data on methodology, population, context, data analysis and authors conclusions were drawn. The data extraction was completed for each study by L.M. and cross checked by K.C. The qualitative data was extracted, including information from illustrative quotes and themes presented by the original author. Each finding was assigned a level of credibility (unequivocal, credible, or not supported) by the reviewers by L.M. according to JBI methodology [33]. This was checked for accuracy by a second reviewer (L.G.‐W.), and any disagreements that arose between the reviewers were resolved through discussion.

### 2.5 Data Synthesis and Integration

This review followed a meta‐aggregative approach to synthesis and integration according to the JBI methodology for qualitative systematic reviews using JBI SUMARI [33]. The author undertook a synthesis of findings that authentically represents the aggregation of data from primary studies. The aggregation process involved combining findings to generate a set of statements that represent that aggregation, through assembling the findings and categorizing these findings based on similarity in meaning. These categories were subjected to a synthesis to produce a single comprehensive set of synthesized findings. All authors were involved in reviewing and checking the accuracy of the final findings.

## 3 Results

**FIGURE 1:**
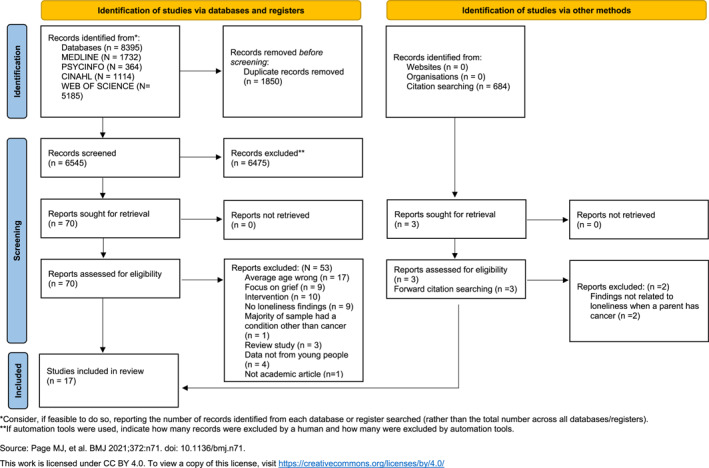
PRISMA flowchart.

Figure 1 shows 8395 articles were identified from the searches, of which 1850 were duplicates and thus deleted before the remaining 6545 articles were imported into Rayyan for screening [34]. Each title and abstract were screened by L.M. with 25% checked by K.C. and L.G‐W. This resulted in the exclusion of 6475 articles. 70 full text articles were then retrieved in full and screened by L.M., L.G.‐W. and K.C. Any disagreements which arose between the reviewers at each stage of the study selection process were resolved with a fourth reviewer (M.D.). 17 articles were included in the review, 2 of which were discovered through citation searching and 15 articles from the database searches.

### 3.1 Study Characteristics

Of the 17 studies included 16 were qualitative and 1 was mixed methods. Of these, 14 studies used cross sectional semi‐structured interviews, 1 retrospective narrative interview, 1 focus group study and 1 serial interview. The papers were published between 2009 and 2024. The studies were carried out in Japan [35], Ireland, [36, 37, 38], Australia [39], the United States [40, 41, 42, 43, 44], Sweden [45, 46, 47] Iran [48], the United Kingdom [49, 50] and Belgium [51]. The papers were published between 2009 and 2024. Details and characteristics of each of the included studies can be seen in Supporting Information S3.

### 3.2 Quality of the Included Studies

Most studies were high quality with 11 of the 17 meeting at least 80% of the JBI Critical Appraisal Checklist for Qualitative Research. The areas where the studies did not meet criteria were due to a lack of detailed reporting about the positionality of the authors and how they may have influenced the data. The checklists for the methodological quality can be found in Supporting Information S4.

## 4 Results

Findings were pooled using JBI SUMARI with the meta‐aggregation approach. 144 findings were extracted with accompanying illustrations. Credibility was assigned to all findings, of which 102 were considered unequivocal, 27 were considered credible, and 15 considered unsupported. Unsupported findings were not reported in the findings of this review. These findings were aggregated into 11 categories, based on similarity of meaning, and then further aggregated into 4 synthesised findings. The full list of extracted findings with accompanying illustrations (Supporting Information S5) and meta‐aggregation tables (Supporting Information S6) are included in the supplementary information.

### 4.1 Synthesised Finding 1: Young People Experiencing Parental Cancer Feel Different and May Not See Peers as a Useful Form of Support

Cancer changes the social fabric of a young person's life. There is a sense of feeling different from peers and more mature due to parental cancer. Time with peers is cut short as the ill parent must be prioritised. Young people encounter difficulty confiding in friends as their peers without lived experience may not understand. In some cases young people's friends reached out to offer support and ask questions about their parent's cancer, but many young people where not ready to accept this offer of support and required alone time to process their situation.

#### 4.1.1 Feeling Different and Not Understood

Young people experiencing parental cancer feel profoundly different from other young people and encounter a lack of understanding from their peers. Young people reported that they felt more mature than their friends because of cancer. Their rapid psychological development left them feeling socially isolated: ‘*In terms of social things and trying to think your‐self into how people feel, taking consideration people, I feel that I've come a lot farther*…’ (D4). Young people explicitly stated that they felt like their friends, who have not experienced parental cancer, would not understand their unique lived experience:‘*my friends don't really understand breast cancer because their parents don't have it. It's nice to tell them but they don't really understand*’ (C6). In some instances when young people attempted to confide in their peers, their peers were emotionally unresponsive to the young person and did not offer them support: ‘…*became completely stiff and started talking about something completely different*’ (F1). This feeling of rejection when discussing parental cancer intensified young people's experience of feeling isolated and different from their friends.

#### 4.1.2 Caring Responsibilities Limit Social Life

Some young people felt that their capacity to socialise was limited because they had to prioritise caring responsibilities at home: ‘*In the past, I was seldom at home, I was with my friends, I didn't think about home and its tasks, but now that my mother is sick, I must be at home all day*…*I have no time for myself*’ (A2). Some of the responsibilities young people prioritise over socialising included looking after younger siblings, cleaning, laundry and driving. This made it difficult to maintain friendships and led to young people being excluded socially: ‘*My friends stopped calling me because I didn't call them. I simply stayed at home all the time*’ (I4). The pressure to stay home sometimes came from the parent who was immuno‐compromised, ‘*she thinks that I'll probably get sick*… *my mom can't*… *risk getting sick*…*with like chemotherapy*’. In other incidences this pressure to stay home was internalised by young person themselves, as the parent's cancer was advanced. This created a feeling of anticipatory grief which meant they placed less value on social activities and time with friends: ‘*Instead of* …*.. going to hang out with your friends*… *stay home with your mom and cuddle with her and hold her hand—* … *she is going to be gone soon, and this is all the time I have left*’ (N5). It is possible that the stage of the parent's cancer may impact and influence the young person's experience of loneliness while their parent is ill.

#### 4.1.3 Not Opening‐Up and Rejection of Peer Support

Young people reported that they avoided confiding in peers and in some cases rejected offers of support from friends. Some young people decided to process parental cancer alone because they felt awkward telling their classmates ‘*You can't go to your friends and say, hey my mom got cancer*’ (C1). Other young people avoided disclosing to peers and rejected support due to a fear of receiving pity from their peers. For others, they avoided talking about cancer because they felt unprepared to face it, which resulted in them feeling emotionally distant from their friends: ‘I *didn't want to talk about it—I just wanted to shove it away*’ (D5). Although some peers attempted to be responsive, young people often rejected this: ‘*I'd made really good friends* …*they [*…*] would come up to my room and knock on the door and [*…*] I just stayed in bed and pretended I wasn't in*’ (L2).

#### 4.1.4 The Importance of Alone Time

Some young people isolated themselves to cope with their parents' cancer and believed that having this time alone was crucial for them to process their feelings. It was necessary to take some space alone when their parent was initially diagnosed: ‘*I had time to myself* …*. so I could think about what was happening* …*. I kind of did this “me time” so I could get used to what was happening*’ (H5). One young person directly stated a desire to be lonely: ‘*I am in my own world, I don't want to speak with anyone, and I want to be lonely*’ (A1) which might be more commonly thought of as solitude. This might suggest that some young people experiencing parental cancer do not differentiate between loneliness and solitude. Aside from needing time for introspection the need for time alone appeared also to stem from feelings of depression: ‘…*before I was always up for going out*…*there were several times* … *I just sat there in my room. I remember …. three days where I just didn't leave my room. Just cried a lot*… *I wasn't myself socially or personality wise*’ (M1). Young people who previously enjoyed the company of friends appear to become more introverted when a parent is diagnosed with cancer and may feel too depressed to socialise.

### 4.2 Young People Experiencing Parental Cancer Are Lonely and Face Overwhelming Negative Emotions Alone

Young people dealing with parental cancer often feel lonely and described struggling to cope with negative emotions alone. There is a strong sense of feeling unrecognised, and powerless against their current set of circumstances.

#### 4.2.1 Dealing With Feelings of Hopelessness and Fear Alone

Young people reported that parental cancer caused them to deal with feelings of hopelessness and fear alone. For example, one young person was alone when they were informed of the diagnosis by text message: ‘*I was alone in my dorm, which was not ideal. I* … *preferred that my parents had told me face‐to‐face*’ (Q16). Even for young people who have family around them there was a deep feeling of loneliness which persists in the presence of others: ‘*Everyone was there at the parting at the hospital, but we were all alone in a way*’ (F3). This suggests that parental cancer creates a sense of fundamental separation from others. Although this young person was aware that their family was going through a very similar experience, there remains a feeling of being alone which cannot be alleviated by the company of other people. Another young person explicitly stated that they felt alone and as though they were the only one experiencing parental cancer: ‘… *I'll just feel alone, like I'm the only one dealing with this*’ (P1). For some young people, there was a sense of anticipatory anxiety about being alone if their parent passes away: ‘… *if my father dies, I'll have nobody else*’ (A3). In other cases, feeling alone and isolated was intensified by COVID‐19 social distancing measures: ‘*Covid made it extra hard* …*in hospital, you can normally still visit*… *during covid. I couldn't see her for 8 weeks. You can video call, but* … *it is not the same*’ (Q18).

#### 4.2.2 Feeling Unrecognised and Unworthy of Help

Several studies reported that young people felt unrecognised as needing support and unworthy of help when their parent has cancer. As parental cancer takes place in the foreground, the young person's needs can go unrecognised in the background. Some young people reported that since their parent had developed cancer they began to feel overlooked and unrecognised: ‘*Nobody asks me what I have been doing? Nobody asks what do you eat? Or what do you wear? Or where have you been?*’ (A4). For other young people feeling unrecognised related more specifically to people not acknowledging how parental cancer has impacted their life: ‘*I don't really think we get recognition*…*they get recognition for being survivors*…*. but we really don't because we are behind the scenes. But we really do a lot*…*one day I was upset because I felt like no one was giving me recognition* …’ (C2). In some instances, young people were encouraged to hide their need for support by their friends: ‘…*they said you should not cry in front of my mom*’ (O6). It is clear that although young people are aware that they feel unrecognised and low, they feel unworthy of support: ‘*there was help for me*… *like my mum gave me the name of a counsellor but* … *I didn't use it because I didn't feel like I deserved it*’ (M8).

### 4.3 Encountering a Lack of Support and Communication at Home

Young people feel a pressure to cope with parental cancer alone even when around family. Parents can become unavailable; siblings may cope differently and there is a dearth of communication about cancer. These circumstances cause young people to feel isolated and in the dark about cancer.

#### 4.3.1 Constrained Family Communication

Several studies reported that young people felt isolated at home due to constrained family communication. In some incidences, this was a result of parents not responding to the young person's information needs, which lead to rumination: ‘ *she just dismisses me really quickly, because she doesn't want it to hurt me, but it's just like the wondering about it feels like it hurts me more than just knowing what's actually going on*’ (J3). In more extreme cases, a young person stated constrained communication can lead to suicidal feelings: ‘*I know a couple of friends that I've had to talk out of suicide and it's because their parents weren't telling them what was going on, they felt isolated*’ (H7). For many young people, they decided to avoid communication to protect their parent: ‘*… you know when something can break easily so you're really careful with it? It was like that*… *you had to think before you spoke a lot about what you were gonna say just in case it upset her*’ (E3). This constrained communication at home appears reciprocal and sometimes extends beyond cancer to avoiding discussing important adolescent life transitions: ‘*She [my mother] did not know what I was hiding, that I really did not feel like going to T. University. I finally told her later. It was like, I was enduring it alone, and I could never say it*’ (02). These circumstances can result in the young person feeling alone when their parent is ill.

#### 4.3.2 Changed Family Dynamics and Support

Young people reported that they felt socially isolated at home as cancer changed the dynamics of their family, leading to reduced familial support. Suddenly, the parent who would normally be the caregiver to the young person was no longer available because of cancer, leaving the young person to cope alone: ‘*I had to work it out somehow myself, and I couldn't call my Mom to talk to*’ (B1). For some young people, the change in family dynamics appears to follow immediately after the diagnosis and may be akin to grief: ‘*After receiving information about the illness it was like Mum died. I counted her out as being there for me*’ (D1). The lack of family support and changed dynamics is further intensified by the well parent prioritising their ill spouse: ‘*One parent was always helping the other parent out with whatever was going on*’ (F10). This shift in family life made young people feel alone and unable to approach their parents This extends not only to parents but also siblings who may prefer to cope on their own: ‘*I didn't talk to my brother about it at all—I knew that I wouldn't get an answer*…*he shut himself out, it just hadn't happened*’ (D3).

### 4.4 Communication and Social Support Protect Young People

Strong family communication, help from teachers or counsellors, and being open to friendship support—especially from peers who have experienced parental cancer first‐hand—are some of the factors that may shield young people from feeling lonely when their parent has cancer.

#### 4.4.1 Communication at Home Is Important

When communication at home is strong, young people feel supported and able to cope with parental cancer. Several studies reported that it was helpful when the healthy parent took time to see how the young person was coping emotionally: ‘*I don't think I was affected as much as other people*…*. Dad was always there to* *support everyone*…*. When Mum went into hospital treatments, Dad was always there to talk to me and my brothers to see if we were going well*’ (H2). It was also reported as helpful to speak to the ill parent about cancer: ‘*I talk to her about (cancer)*… *and it helps*’ (K2). One young person stated that when family communication is strong, it is not necessary to receive external support: ‘*We didn't need to go somewhere else to talk, because we have always had each other, and we can talk about anything*’ (I1). Having a sibling to confide in was also very helpful*:* ‘*I have a sister* …*. She's my best friend. She's in the exact same situation* … *she's only a year older than me*… *we understand each other completely. I can really tell her everything*’ (Q10).

#### 4.4.2 Support From Adults Outside of the Family

Young people report that their feelings of aloneness can be alleviated by support from trusted adults outside of their family. Young people appreciated when adults offered them some support: ‘… *it was my advisor, it somehow seemed like they knew what I was going through, and they listened to what I had to say*… *they would notice I had something I could not say to others, and they would talk to me. It was as if I was not alone*’ (O3). It is also useful for young people to speak to teachers at school about how they are feeling*:* ‘*My religion teacher and the school chaplain like I'd have chats every week, like she'd spot me, and she would be like “(name), would you like to come for a chat?” [*…*] they were great* *support*’. For some young people, they decided to get professional support from a counsellor. This was highlighted as being supportive and it allowed the young person to fully express their emotion to someone they considered objective: ‘*it was really the best thing for me because I just cried and talked about everything*… *it really did help*…*to talk to someone that was really objective to the situation*’ (P7). Talking to adults about parental cancer prevents young people ruminating alone: ‘*releases the stress or like the thoughts that are in my head*’ (P5). It was also helpful when medical professionals acknowledged that the young person was impacted too*:* ‘*I just like it when they [the medics] ask me how I'm doing when I'm at the hospital. For me, a sincere “how are you is already a sign of acknowledgement*…”’ (Q7).

#### 4.4.3 The Importance of Friendship and Shared Experience

Young people reported that friendship was an important when dealing with parental cancer. Young people who had responsive friends reported that ‘*a friend can get it just right*’ (E4). In circumstances where the young person became overwhelmed with emotions, they felt relieved when a friend noticed and responded with support: ‘*I was in the middle of class, and [friend] just stood up and gave me a hug*… *it made everything better*’ (N3). Young people also particularly valued support from other young people who had experience of parental illness: ‘*I've been* *supported by friends who have been through similar experiences*’ (E2). There was a sense that peers with lived experience of parental illness understand the young person, making it easier to open up: ‘*I have many friends whose parents also have had cancer, and then we share information* … *it's totally different because we can relate*’ (J13). For other young people who lacked the support of peers with lived experience of parental illness, they expressed that a peer support group would help: ‘ *if you went to the group with the same people and then you build relationships*… *We would come*’ (C4). Another young person similarly remarked that ‘*I just want someone to tell me it will be ok*… *someone who has been through the process with their parent*’ (C3).

## 5 Discussion

This qualitative systematic review is the first review to synthesise experiences of loneliness in adolescents experiencing parental cancer and has contributed to a comprehensive and novel understanding of loneliness in this population. This review only identified 17 papers which included findings relating to loneliness, meaning the evidence base regarding adolescent loneliness when a parent has cancer is reasonably small. Although a small number of studies were included, the meta‐aggregation of these papers resulted in four synthesised findings. This meta‐aggregation found that young people experiencing parental cancer experience loneliness and deal with overwhelming emotions alone. Young people's experiences of loneliness are often intensified by a lack of understanding from peers and constrained communication at home. Potential protective factors against loneliness include strong family, friendship and school support, and peers who have lived experience of parental cancer.

### 5.1 Friendship and Peer Support

This review revealed that young people experiencing parental cancer often do not see their friends as a source of support, which contributed to feelings of loneliness. This is consistent with previous reviews on parental cancer [20] which ascertained that young people's most reported unmet need when a parent has cancer is support from friends. A significant barrier which prevented young people from seeking friendship support was feeling ‘different’ from their peers. Young people experiencing parental cancer have a unique family situation which set them apart from their peers by comparison. Similar themes are present in extant qualitative literature on adolescent loneliness. Lonely youth report feeling fundamentally ‘different’ to others, with this feeling often stemming from social comparison [52, 53]. In the case of adolescent loneliness more generally, youth could not always identify why they felt different to peers [52]. Contrastingly, this review revealed that adolescents experiencing parental cancer can articulate ways in which their life is different from peers and are aware of how this can impact relationships and connectivity with other young people. Young people in this review were reluctant to discuss their experiences with peers who have healthy parents, in case their experiences were not understood. This led to young people preferring to reject peer support and isolate themselves in order to cope with their parent's cancer.

### 5.2 Home Life and Family Communication

Young people experiencing parental cancer decided to prioritise family and home life when their parent was ill, which resulted in less value placed upon socialising with peers. A narrative review of loneliness among cancer caregivers [30] reveals that as cancer care moves towards a more community‐based setting, there has been an increase of informal carers such as family and friends supporting cancer patients. This can limit informal caregivers' ability to socialise and led to loneliness and loss of friends [54, 55] Indeed, young people in the current review also reported a loss of friends who stopped calling them when they noticed the young person staying home more often. In particular, some young people put a pressure on themselves to stay home as their parent's cancer was advanced. With the young person experiencing anticipatory grief, they were unable to focus on their social life or friends and instead isolated themselves from those support networks to make the most of the time they had left with their parent. For young people whose parent's cancer was curative, they still felt the future was uncertain and they too reported that their priorities had shifted away from peers and socialising towards family life in case their parent passed away. With pressure to stay home and the challenge maintain contact with friends when a family member has cancer, it is unsurprising that young people struggle with loneliness.

The wider literature on parent's experience of cancer whilst they have dependent children indicates that parents are keen to maintain a normal family life, even when their diagnosis is advanced [56]. For example, parents report a pressure to look and behave as ‘normally’ as possible to avoid distress to their children [57]. Despite parents' effort to maintain normalcy, young people report that periods of hospitalisation and treatment lead to changes and disruption in home life such as less chance to partake in extracurriculars and changes to routines [58]. There is evidence to suggest that when a parent has cancer, the young person's evaluation of their mental health can differ from the parent's evaluation, which may lead to instances where a parent can underestimate their child's mental and emotional issues [57]. The findings of this review indicate that cancer impacts the entire family and can lead to changed family dynamics at home. In some families, the well parent must become a carer for the sick parent, which may lead to a situation where parents could be less available to their children. These circumstances caused deep feelings of loneliness for young people and suggest that despite parent's best efforts to maintain normalcy, cancer can still cause natural changes to the family dynamic.

The results of this review indicate that young people felt lonely due to constrained communication at home. Young people in this review believed their parents were uncomfortable discussing cancer and avoided conversations about it. Young people also reported that they too felt uncomfortable initiating conversations about cancer out of concern for their parents' feelings. Previous systematic review evidence highlights that young people report unmet informational needs about cancer and that constrained communication is experienced both young people and parents [20]. Our findings suggest that constrained communication left youth feeling isolated and ruminating about cancer. Rumination is thought to be a key feature of loneliness which is intensified by having no one to confide in [59]. It is likely that young people experiencing parental cancer may benefit from communication from healthcare professionals and their parents, to prevent them ruminating alone, in the absence of information.

### 5.3 Clinical Implications

For young people to access emotional support their parent has cancer, they may identify their own need for help and seek support for themselves by speaking to a teacher or healthcare professional for example. More commonly, however, young people might rely on their parents to recognise that they need help, and to respond by referring them an appropriate pathway of formal support [57]. In the absence of communication about parental cancer and the emotional impact of this on the family, young people may be more vulnerable towards experiencing issues such as loneliness without formal support. This review highlights that conversations at home around cancer protect young people against experiencing loneliness. Of course, it is important to recognise that is very difficult for parents to discuss cancer with their children, especially when they may not feeling emotionally ready. Evidence shows that parents may find it challenging to speak their children about cancer, especially in a way that is child‐friendly and will not generate more anxiety in the young person [60]. Parents' also highlight that it is particularly difficult to talk about prognosis and death with their child [60]. It is essential that healthcare professionals encourage and empower parents talk about their cancer with their young people at home and provide them with the tools and resources to best approach these discussions as a family. As parents who are recently diagnosed with cancer need support and encouragement to communicate openly with their family [58], ideally, healthcare professionals should ask individuals with cancer if they have children early on. Once this is identified, professionals are better placed to encourage parents to have conversations about cancer with their young people. Conversations from the point of diagnosis with young people about cancer would give parents and professionals an opportunity to detect loneliness and difficult emotions in young people early, before more complicated outcomes arise.

Young people in this review felt a sense of disconnection from their peers who lacked lived experience of parental cancer and found it challenging to discuss cancer with their friends. This resulted in feelings of loneliness. The young people highlighted that they valued friendships based on shared experiences of parental cancer, and that a peer support group would be helpful. There is a dearth of evidence on the efficacy of peer support groups for young people experiencing parental cancer. However, findings from an adventure‐based support group for young reported a reduction in young people's self‐reported loneliness [61]. The young people valued being part of a community of peers with shared experiences and felt better supported [61]. Healthcare professionals should ensure that families who have dependent young people are referred to services were young people can access peer support, and parents are made aware of any programmes which may be available to their young person. Within the United Kingdom, there is currently a lack of help for young people experiencing family illness and current provisions are thought to not be appropriately supported by policy intervention [62]. One organisation which is attempting to combat this issue is Hope Support Services [62]. This is a service based in England that provides support to young people aged 5–25, when a family member is diagnosed with a life‐threatening illness. Hope report that 74.3% of their referrals are from young people experiencing familial cancer, and one of the most prevalent issues young people report with is loneliness and isolation [62]. Hope Support Services offer excellent peer support groups to young people indicating that there are some excellent avenues of support available for young people experiencing loneliness when their parent has cancer, which parents could be made aware of.

### 5.4 Strengths and Limitations

The use of a Joanna‐Briggs (JBI) meta‐aggregation design enabled the synthesis of the best available evidence in relation to how adolescents experience loneliness when a parent has cancer. JBI is a robust methodology which aims to aggregate the findings of previous authors as opposed to reanalyse them. This minimises the risk of bias when synthesising qualitative findings. The use of this methodology has resulted in a review which provides a robust and rigorous insight into how adolescents experience loneliness when a parent has cancer. The main weakness of this review was the dearth of studies which consider adolescent loneliness when a parent has cancer. Only Karlsson et al. [22] discusses loneliness as the principle finding when interviewing young people regarding parental cancer. However, the other included articles did acknowledge instances of loneliness less explicitly. Unfortunately, several papers mentioned the word loneliness or isolated explicitly in their findings and discussion but provided no quotation to evidence this. Due to JBI guidelines these findings were considered unsupported and not included in the manuscript.

## 6 Conclusion

This review has identified how adolescents experience loneliness when their parent has cancer, as well as providing insight into the perceived factors which might influence loneliness in this population. When the papers included in this review are taken individually, themes and patterns of loneliness often emerged implicitly throughout the data. However, when these papers were subject to a meta‐aggregation a clear theme of loneliness emerged. The literature base on adolescent loneliness when a parent has cancer is understudied. However, the results from this review evidence that adolescents do experience loneliness when their parent has cancer. Future research should aim to address the lack of research by interviewing this population of young people directly with the view of developing a more comprehensive understanding of their lived experience.

## Author Contributions

All authors (L.M., K.C., M.D., J.G. and L.G.‐W.) contributed to the conception and design of the work, the data collection and analysis. L.M. drafted the work, and all authors approved the submitted version and agree to be personally accountable for the author's own contributions and to ensure questions related to accuracy or integrity of any part of the work, even ones in which the author was not personally involved, are appropriately investigated, resolved, and the resolution documented in the literature. All authors read and approved the final manuscript.

## Conflicts of Interest

The authors declare no conflicts of interest.

## Supporting information

Supporting Informarion S1

Supporting Informarion S2

Supporting Informarion S3

Supporting Informarion S4

Supporting Informarion S5

Supporting Informarion S6

## Data Availability

Data sharing not applicable to this article as no datasets were generated or analysed during the current study.
