# Supplementary material for: Adolescent Loneliness When a Parent Has Cancer: A Qualitative Systematic Review
Source: Psychooncology. 2025 Apr 8;34(4):e70148. doi: 10.1002/pon.70148 (PMC11979319; doi:10.1002/pon.70148)
Supplement: Supplementary file 2 — Supporting Informarion S2 [file PON-34-e70148-s004.docx]

**Supplementary Material 2: Medline Search**

**Database:**
Ovid MEDLINE(R) ALL <1946 to May 15, 2023>

| **#** | **Query** | **Results from 16 May 2023** |
| --- | --- | --- |
| 1 | cancer.mp. | 2,163,117 |
| 2 | malignan*.mp. | 689,239 |
| 3 | Neoplasms/ | 497,225 |
| 4 | offspring.mp. | 86,099 |
| 5 | son.mp. | 27,350 |
| 6 | daughter.mp. | 20,870 |
| 7 | Child/ | 1,905,117 |
| 8 | unmet needs.mp. | 9,320 |
| 9 | distress.mp. | 169,687 |
| 10 | Bereavement/ | 6,629 |
| 11 | bereave*.mp. | 12,047 |
| 12 | coping.mp. | 71,125 |
| 13 | 1 or 2 or 3 | 2,777,435 |
| 14 | 4 or 5 or 6 or 7 | 2,025,003 |
| 15 | 8 or 9 or 10 or 11 or 12 | 251,723 |
| 16 | 13 and 14 and 15 | 2,154 |
| 17 | limit 16 to (english language and yr="2007 -Current") | 1,478 |

cancer.mp.
malignan*.mp.
Neoplasms/
offspring.mp.
son.mp.
daughter.mp.
Child/
unmet needs.mp.
distress.mp.
Bereavement/
bereave*.mp.
coping.mp.
1 or 2 or 3
4 or 5 or 6 or 7
8 or 9 or 10 or 11 or 12
13 and 14 and 15
limit 16 to (english language and yr="2007 -Current")
