# Supplementary material for: Adolescent Loneliness When a Parent Has Cancer: A Qualitative Systematic Review
Source: Psychooncology. 2025 Apr 8;34(4):e70148. doi: 10.1002/pon.70148 (PMC11979319; doi:10.1002/pon.70148)
Supplement: Supplementary file 3 — Supporting Informarion S3 [file PON-34-e70148-s003.docx]

| **Study**  Supplementary Material 3: Participant Characteristics | **Design and methodology** | **Phenomena of interest** | **Setting** | **Participant characteristics and sample size** | **Description of main results relating to loneliness** |
| --- | --- | --- | --- | --- | --- |
| Azarbarzin, Malekian & Taleghani (2016).  Iran | Semi-structured interviews  The data was analysed using constant comparative analysis. | The purpose of this qualitative study was to explore Iranian adolescents’ experiences when living with a parent with cancer. | Cancer centres, an oncologist’s clinic, a chemotherapy clinic and public places such as a park and the home of patients | Sample: 16 Iranian adolescents’ living with a parent with cancer.  Age: Ranged from 12-20, mean age 16).  Gender: 10 females and 6 males.  Ethnicity: Iranian | This research showed that Iranian adolescents' experiences were largely consistent with other adolescents' experiences of parent cancer. The young people in this sample reported feelings of loneliness and social isolation, sometimes choosing to isolate themselves from the outer world in order to process cancer. The young people felt no one checked in on how they were doing and felt they now must stay home and do chores instead of going out with friends |
| Clemmens, (2009)  United States of America | Semi-structured interviews  Study was guided by Van Manen’s (1990) phenomenologic interpretive paradigm. | To explore adolescents’ experiences of living with mothers with breast cancer. | Clinics, office spaces and participant homes. | Sample: 11 adolescents whose mothers were diagnosed with breast cancer within a year prior to the study.  Age: 13–19 years old  Gender: 5 males and 6 females.  Ethnicity: The participants identified themselves as being Caucasian, African American, or Hispanic. | All participants expressed they were struggling with feelings of anxiety. Life with a parent who has cancer is challenging, and there is pressure to take on a domestic role at home. Young people turned toward themselves and searched for solitude in music and alone time, but also struggling with feeling as though their mother was now absent. Open communication was important to participants. |
| Davey, Tubbs, Kissil & Niño (2011)  United States of America | Three focus groups. which used a semi-structured discussion format.  A content analysis method was used. | To explore African American youth and how they cope with the diagnosis and treatment of parental breast cancer. | University | Sample: 12 young people experiencing parental breast cancer.  Age: Aged 11-18, mean age 14.5.  Gender: 2 male, 9 female.  Ethnicity: African American | Young people in this study tried to not think about cancer and used distraction techniques to shield themselves. The young people struggled with feelings of isolation; they preferred to keep emotions to themselves and found it difficult to speak to friends about parental cancer, especially if friends did not share this life experience. Young people felt unrecognised and struggled with feelings of uncertainty, they felt that a 'teen group' of other young people with lived experience would be a great resource for young people experiencing parental cancer, pointing to the value of peer support. |
| Dehlin & Reg, (2009).  Sweden | Semi-structured interviews.  The analysis was inspired by Starrin and Svensson (1994). | To explore adolescents’ experiences in the serious illness and death of a parent. | Hospital | Sample: Five adolescents who were 14–17 years of age when one of their parents died. According to the adolescents, the period of illness before the death was between 4 months and approximately 2 years. Two of the adolescents were siblings.  Age: 16–18-year-olds  Gender: 2 female and 3 male.  Ethnicity: Undisclosed | Young people struggled with existential questions and the justice of life after their parents became ill. Support from family and friends was important and provided some relief. The adolescents prioritised staying at home with their ill parents to make the most of limited time together, and to do domestic chores. Some young people avoided talking about the illness to cope and began to 'count out' their ill parents even before they passed. A significant theme called I Bear This Alone emerged from the findings. Ultimately, the young people felt alone, lonely and alienated. They did not reach out to their parents or siblings for support. The experience of loneliness was made stronger by relationships with friends who never asked them about the ill parent. Young people feel misunderstood by their peers and singled out at school because of parental cancer. There also is a sense of feeling much more mature than peers and changed by the experience of parental cancer. |
| Finch & Gibson (2009).  United Kingdom | Semi-structured interviews.  Data analysed using interpretative phenomenological approach. | To explore how young people experience learning about their parent’s cancer diagnosis. | London Cancer Centre or participants' home. | Sample: Seven adolescent participants experiencing parental cancer.    Age: Aged 14-18, mean age 16.3  Gender: 4 males and 3 females  Ethnicity: Not disclosed | Some young people struggle with feeling isolated in their experiences and begin to experience existential feelings. Young people hid their emotions at home and one participant stated his family did not communicate about his father's cancer leading to feelings of isolation. Support from friends was important, especially those with similar experiences. Some young people reported teachers at school only asked about parental cancer at the beginning and then forgot to check in on the young people. |
| Fujimoto & Kanda (2023).  Japan | Semi-structured interviews.  Analysed using Krippendorff’s (2012) content analysis design. | To explore the experiences of adolescent daughters who are beginning to live with their mothers’ cancer diagnosis. | Unspecified. | Sample: 14 adolescent daughters of mothers who were diagnosed with breast cancer.  Gender: 14 Females  Age: The mean age was 17.01, ranging from 15 to 19 years old.  Ethnicity: Not disclosed | Young people in this study reported that they struggled with feelings of anxiety even when they tried to shield themselves from cancer. The daughters felt they had to deal with life transitions alone and reported that parental cancer impacted their social and school life. When these young people were supported by relatives and school staff they felt less alone and were able to cope better. |
| Karlsson, Andersson & Ahlström (2013).  Sweden | Retrospective narrative interviews.  The narratives were analysed with content analysis. | To explore young adults’ own perspectives on the experience of having a parent who developed cancer when the young adult was an adolescent. | Participant’s own homes, the university, and hospital. | Sample: Six young people who experienced during their adolescence (13-19 years) a parent being diagnosed with cancer.  Age: 20-26 years old, mean age was 20.8 years old. The average during the onset of parental illness was 15.  Gender: Not disclosed.  Ethnicity: Not disclosed. | Loneliness was the main finding of this paper. Loneliness was described both as physical loneliness and as a feeling of being alone in their experience and there was a profound feeling of others not understanding what they were going through. The loneliness that the young adults experienced permeated their lives and was expressed not only in terms of distance but also as an undertone when expressing closeness. |
| Marshall, Fearnley, Bristowe & Harding (2022).  United Kingdom | Sem-structured interviews.  Data was analysed using thematic analysis | To explore the perspective of children on living with a parental life-limiting illness. | Unspecified | Sample: Young people experiencing a with parental life-limiting illness. Majority of the sample was experiencing parental cancer.  Age: Aged 6-17  Gender: 16 male and 16 female participants.  Ethnicity: Asian or Asian British = 1 (3%) Black, Black British, Caribbean or African = 5 (16%) Mixed or multiple ethnic groups = 4 (12.5%) Other ethnic group = 4 (12.5%) White = 18 (56%) | Young people in this study had to prioritise helping at home, leaving less time to spend socialising or with peers. There was an anxiety that if they left home, their parent may need their support and be unable to reach them. It was difficult to hear peers at school discuss things such as going on holiday when the young person was unable to due to parental illness. This made them feel left out or different. Young people also reported not wanting to discuss parental cancer with friends at school. The participants felt defenceless against the impact and shock of their parents being ill. |
| Maynard, Patterson McDonald & Stevens (2013).  Australia | Semi-structured interviews.  Phenomenological thematic analysis was utilized to analyse the data. | To explore what is helpful for young people who have a parent diagnosed with cancer. | Unspecified | Sample: 15 adolescents who had a parent diagnosed with cancer within the last 5 years.  Age: Mean = 15.9 years, ranging from 14 to 22 years old.  Gender: 9 females and 6 males.  Ethnicity: Not disclosed. | Parents being present to answer questions about cancer helped young people feel supported, however, young people still struggled with feeling guilty for discussing their feelings. The adolescents urged parents to not withhold information as it can lead to feelings of isolation, however also highlighted the need for space away from cancer physically and psychologically, with time alone being important. Friendship was an important source of support, particularly from peers with lived experience of parental cancer. |
| Melcher, Sandell & Henriksson (2015)  Sweden | Serial interviews.  Interviews were analyzed using qualitative content analysis. | To explore teenagers living in a family with a seriously ill and dying parent. | Specialist palliative care units | Sample: 10 adolescents living in a family with a seriously ill and dying parent. Majority of the parents had an advanced cancer diagnosis.  Age: Participants were aged 14-19 years old.  Gender: 7 males and 3 females.  Ethnicity: Undisclosed | Some young people struggled with feeling alone as their parents did not communicate with them about cancer. There was a shift of domestic responsibility onto the young person and young people felt they had to hide their emotions from their parents. Young people spent time alone as a result of parental cancer and experienced loneliness. This was alleviated for some participants by strong family support and having a normal day to day life at school. |
| Phillips & Lewis (2015).  United States of America | Semi-structured interviews.  Analysed using a hermeneutic phenomenological approach | To explore adolescents experiencing parental cancer. | Family’s home or at another convenient private location identified by the subjects. | Sample: 10 adolescents who had a parent with advanced cancer.  Age: Age ranged age from 11 to 15 years (mean = 13.6 years, standard  deviation = 1.4 years).  Gender: Five females and two males.  Ethnicity: Not disclosed. | Young people felt it was difficult to deal with uncertainty and a lack of control over cancer. They felt alone and as though they were the only person dealing with parental cancer, and experienced minimal support from outside of their family. Some young people found it helpful to talk to friends while others reported a lack of understanding from peers. |
| Phillips & Lewis (2015).  United States of America | Semi-structured elicitation interviews.  Inductive content analysis was used to analysis. | To explore the experience of adolescents living with a parent with advanced cancer from the adolescents’ perspective as well as to describe the impact of advanced parental cancer on adolescent functioning. | Face-to-face and in-home interviews. | Sample: 7 adolescents from six families currently living with a parent diagnosed with advanced or metastatic cancer stage 3 or above.  Age: Age ranged from 11-15 years old with a mean age of 13.6.  Gender: Five female and two male adolescents  Ethnicity: Caucasian. | Young people struggled with their parents becoming unavailable due to hospital appointments or changes in their personalities attributed to medication or illness. Cancer limits young people's lives - medical bills are expensive, there is a risk of making the immunosuppressed parent ill when socialising and there is a pressure to stay home to provide support. Talking to others such as friends or siblings is helpful, especially from peers with lived experience. |
| Rodriguez (2019).  Ireland | Semis structured.  The analysis was secondary thematic analysis. | To explore the communication patterns in adolescents that faced maternal cancer. | Face-to-face and in-home interviews. | Sample: 15 adolescents  Gender: 10 females and 5 males.  Age: Mean age was 17, ranging from 16-20 years old  Ethnicity: Not disclosed. | It was helpful for young people to communicate about cancer; however, the adolescents were selective about whom they shared their feelings. It was difficult to deal with their mother being less available to support them as she dealt with cancer herself and some young people found it hard to be away from home at university during this time. Adolescents confided in friends but struggled with worrying about how their peers may react with pity. |
| Rodriguez, Dolan, Kerin & Groarke (2022).  Ireland | This qualitative study was a secondary data analysis of semi-structured interviews.  Data analysed using a secondary content analysis. | To explore the unmet needs of adolescents experiencing maternal cancer in Ireland. | Face-to-face and in-home interviews. | Sample: 15 adolescents completed qualitative interviews as part of a larger study that explored the experience of adolescent adjustment to maternal cancer.  Age: Aged 14–20-year-old  Gender: 5 male, 10 female  Ethnicity: Undisclosed. | Young people reported wanting to be kept within the informational loop of cancer. Some young people reported that they did not talk about cancer because they were scared of the reaction other people would have or their families were too sensitive to discuss it. They reported isolating themselves to their room to process difficult emotions and being kept busy at home doing domestic chores. The participants reported that friends forgot to check on them and they would prefer to talk to those with a similar experience and sometimes avoided chatting to friends who haven't experienced parental cancer. |
| Rodriguez, Groarke, Dolan & MacNeela (2018).  Ireland | This study was mixed method. It included a quantitative survey with 40 adolescents, with 15 completing semi-structured interviews.  Three participant transcripts were selected for IPA. | To explore adolescent experiences of maternal cancer. | Interviews were face-to-face. | Sample: This analysis is focussed on 3 female adolescents with experiences of maternal cancer.  Age: Aged 17-20  Gender: 3 females.  Ethnicity: Undisclosed. | Some participants reported feeling unable to express themselves at home and holding back their feelings from their ill mother. Although support from friends was appreciated the young people were selective in who they told and did not like to be checked in emotionally too often. One of the participants had to leave university as she felt too overwhelmed, while another felt set apart from her peers at university due to cancer. |
| Sheehan, Mayo, Christ, Heim, Parish, Shahrour & Draucker (2016).    United States of America | Semi-structured interviews.  The data was analysed using grounded-theory methods. | To explore the coping strategies that adolescents employ to manage the stressors they experience in the final months of their ill parent’s life and shortly after their death. | Participants’ homes or in a private room at the hospice facility. | Sample: The sample included 26 families of adolescents with a parent receiving care in a large hospice. Parents or guardians (n=14) who were ill, well parents/guardians (n=17) and adolescents (n=30) prior to parental death, and families (n=6) post parental death.  Age: Adolescents were aged 12-18, mean age was 15.  Gender: 12 males, 18 females.  Ethnicity: Caucasian n=22 (73%) African American n=5 (17%) Mixed race n=2 (7%) No response n=1 (3%) | Young people reported pressure to be around to deal with domestic chores. The adolescents reported that they mostly preferred to not discuss their parent’s illness or allow their friends to see how sick their parent was. Some of the young people became anxious when away from home attempting to engage in social activities like school football game. |
| Tulpin, Scherrens, Van Driessche, Verhofstadt, Kreicbergs, Goubert & Beernaert  (2024)  Belgium | Semi-structured interviews.  The data was analysed using conventional Qualitative Content Analysis (QCA). | To gain insight into the perceived support needs of AYAs who have a parent with cancer, and the perceived factors facilitating or hindering their use of support. | Participants’ homes or online. | Sample: 17 AYAs who have a parent with cancer of any type and stage.  Age: Aged 15-26, with a mean age of 18.8 years old  Gender: 11 females, 6 males.  Ethnicity: Not provided. | Young people in this study emphasized the importance of receiving honest and clear information. Sharing news of a parental cancer diagnosis with peers was difficult. Some young people felt they needed time to process this privately. Young people preferred to ask the healthy parent for information to avoid upsetting the ill parent. Family, friends, peers (i.e., people their age who also have a parent with cancer) and school staff were identified as important informal sources of support. Young people did sometimes enjoy time with friends but reported that they felt guilty for not being at home. |
