# Supplementary material for: Adolescent Loneliness When a Parent Has Cancer: A Qualitative Systematic Review
Source: Psychooncology. 2025 Apr 8;34(4):e70148. doi: 10.1002/pon.70148 (PMC11979319; doi:10.1002/pon.70148)

## **Supplementary Material 4: Methodological Quality of Included Studies**

### **Table 1 – The Critical Appraisal of Included Studies**

***05/08/2024***

### Critical Appraisal Results

| **Citation** | **Q1** | **Q2** | **Q3** | **Q4** | **Q5** | **Q6** | **Q7** | **Q8** | **Q9** | **Q10** | **%** |
| --- | --- | --- | --- | --- | --- | --- | --- | --- | --- | --- | --- |
| Azarbarzin M, Malekian A, Taleghani F. 2016. | U | Y | Y | Y | Y | N | Y | Y | Y | Y | 90% |
| Clemmens DA. 2009. | Y | Y | Y | Y | Y | Y | N | Y | Y | Y | 90% |
| Davey MP, Tubbs CY, Kissil K, Nino A. 2011. | U | Y | Y | Y | Y | Y | N | Y | U | Y | 70% |
| Dehlin L, Martensson L. 2009. | Y | Y | Y | Y | Y | N | Y | Y | Y | Y | 90% |
| Finch A, Gibson F. 2009. | Y | Y | Y | Y | Y | N | Y | Y | Y | Y | 90% |
| Karlsson E, Andersson K, AhlstrÃ¶m BH. 2013. | U | Y | Y | Y | Y | N | N | Y | Y | Y | 70% |
| Marshall S, Fearnley R, Bristowe K, Harding R. 2022. | Y | Y | Y | Y | Y | N | N | Y | Y | Y | 80% |
| Maynard A, Patterson P, McDonald FEJ, Stevens G. 2013. | U | Y | Y | Y | Y | U | Y | Y | N | Y | 70% |
| Melcher U, ell R, Henriksson A. 2015. | U | Y | Y | Y | Y | N | N | Y | Y | Y | 70% |
| Phillips F. 2015. | Y | Y | Y | Y | Y | Y | Y | Y | Y | Y | 100% |
| Phillips F, Lewis FM. 2015. | N | Y | Y | Y | Y | Y | Y | Y | Y | Y | 90% |
| Rodriguez L, Dolan P, Kerin M, Groarke A. 2022. | N | Y | Y | Y | Y | N | N | Y | Y | Y | 70% |
| Rodriguez L, Groarke AM, Dolan P, MacNeela P. 2018. | Y | Y | Y | Y | Y | Y | U | Y | Y | Y | 90% |
| Sheehan DK, Mayo MM, Christ GH, Heim K, Parish S, Shahrour G, et al. 2016. | Y | Y | Y | Y | Y | Y | Y | Y | Y | Y | 100% |
| Keiko Fujimoto KK. 2023. | Y | Y | Y | Y | Y | Y | Y | Y | Y | Y | 100% |
| Leonor Rodriguez. 2019. | U | Y | Y | Y | Y | Y | Y | Y | Y | Y | 100% |
| Tulpin  2024 | U | Y | Y | Y | Y | N | U | Y | Y | Y | 70% |
| % | 47.05 | 100.0 | 100.0 | 100.0 | 100.0 | 47.05 | 52.94 | 100.0 | 88.23 | 100.0 |  |

**Table 2: The JBI Critical Appraisal Checklist for Qualitative Research**


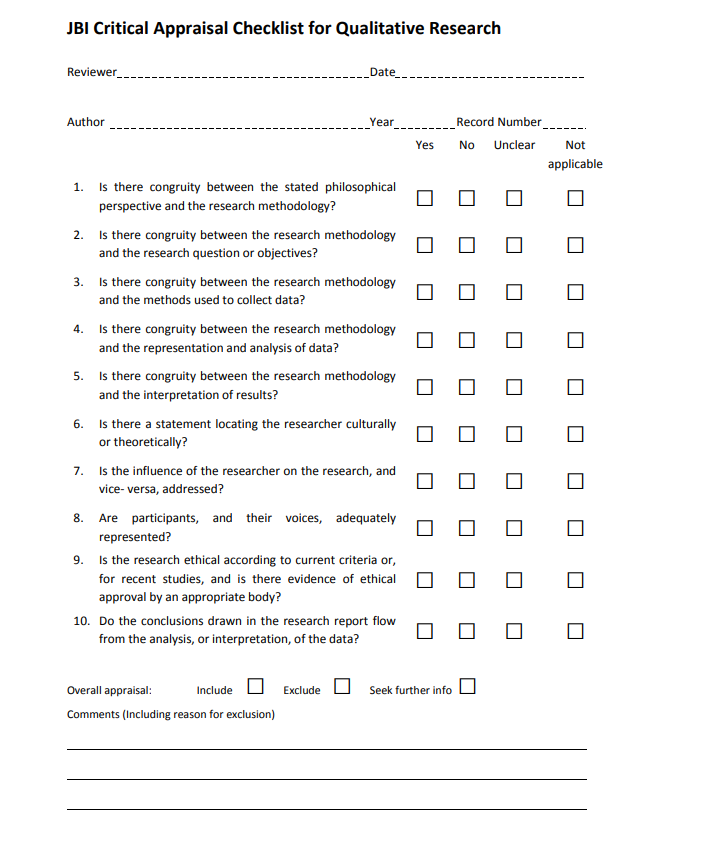

Supplement: Supplementary file 4 — Supporting Informarion S4 [file PON-34-e70148-s005.docx]
