# Supplementary material for: Adolescent Loneliness When a Parent Has Cancer: A Qualitative Systematic Review
Source: Psychooncology. 2025 Apr 8;34(4):e70148. doi: 10.1002/pon.70148 (PMC11979319; doi:10.1002/pon.70148)
Supplement: Supplementary file 6 — Supporting Informarion S6 [file PON-34-e70148-s006.docx]

## **Supplementary Material 6: Meta-Aggregation Tables**

**Table 1: *Synthesised Finding 1: Young people experiencing parental cancer feel different and may not see peers as a useful form of support.***

| **Findings (n=46)** | **Categories (n=4)** | **Synthesised Finding** |
| --- | --- | --- |
| (C6) Some young people display an awareness that friends do not have shared experiences of parental cancer. Although it is nice to reach out for support there is a feeling that friends will lack insight. [U] *"my friends don't really understand breast cancer because their parents don't have it. It's nice to tell them but they don't really understand”.*  (D4) Parental cancer can cause young people to feel more mature than their peers who have not experienced this. It leads to them being more empathetic. [U] “*In terms of social things and trying to think your-self into how people feel, taking consideration people, I feel that I’ve come a lot farther than [my friends]—umm, I have a higher grade in that."*  (F1) Friends can reject conversations about parental cancer when the young person brings it up by becoming uncomfortable or changing the subject. [C] *"I only had to mention that he...so did he or like that, from everyday life, but I couldn’t, they became completely stiff and started talking about something completely different instead."*  (G6) Young people experiencing parental cancer might feel quite different from peers as their ill parent is less physically able compared to healthy parents. This means they are unable to go on holiday like their peers. [C] ‘*when I’m at school and like. . .people are talking about stuff that they do on the holidays with their mum. . .erm. . .and I can’t do that’*    (J11) There is a sense of feeling different from peers due to financial issues stemming from cancer. [C] *"Because of all the doctor bills, I mean, we’re not that financially well off, and I mean it’s kind of frustrating going to my school. Everyone has got a lot of money, and we don’t have any money because of my dad’s expensive bills. So I mean that can be pretty frustrating. "*  (J14) The experience of parental cancer can cause the young person to feel more mature than other young people due to the amount of responsibility they have been exposed to. [U] *"I’m more mature than I would have been because I, at a young age, I learned how to like take care of my mom more and, like, take on responsibilities most younger kids wouldn’t have"*  (K9) It is difficult to see peers with healthy or living parents. The comparison between life with parental cancer and peer's lives feels depressing. [U] *"I feel really bad cause my dad won’t live as long as other dads will. I worry when my dad is gone. I’ll really miss him and when everyone else has dads and I don’t, it will be really depressing”.*  (K4) Young people can encounter a lack of understanding from peers about parental cancer. [U] *"When I go talk to my friends ... and I tell them ... they don’t really understand to have a parent with cancer .... Mostly because I talk to my friends about almost all my problems and they don’t know what I’m really talking about."*  (M2) Friends can forget to check in about parental cancer as time passes on from the diagnosis. [U] "*I feel like at the start people were worried but then, I’d never blame them for it but because it is not a constant, not within their own family and it’s not on their mind all the time, they can forget about it(...)"*  (Q13) Young people struggle to open with their peers as they find that friends may misunderstand diagnostic information about cancer (C): *“What’s hard for me is that cancer is such a general term. If you tell your friends something about it, like a good result, they assume the cancer is completely cured. While that isn’t the case. The news may be positive, but it’s something that will never go away. That makes me feel misunderstood.”*  (Q19) It is a good thing friends have not had to experience parental cancer, but this can lead to the young person feeling misunderstood (C): *“There are very few people I can talk about it with properly. Because no one understands. And it’s a good thing my friends don’t understand. That they haven’t been through it. But sometimes I do need someone who’s also experiencing this, someone who’s in my shoes.”* | 1.1 Feeling different and not understood. | Cancer changes the social fabric of a young person’s life. There is a sense of feeling different from peers and more mature due to parental cancer. Time with peers is cut short as the ill parent must be prioritised. Young people encounter difficulty opening up to friends as they are cognisant that peers without lived experience may not understand. Even where friendship support is available, the young person often does not feel ready or comfortable to accept this. In some cases, this is because the young person requires time alone to process their parent’s cancer. |
| (A2) Prior to parental cancer there was more time for young people to focus on spending with their friends. Now their time is more focused on domestic tasks, and there is a lack of time to focus on their own personal life. [U] *“In the past, I was seldom at home, I was with my friends, I didn’t think about home and its tasks, but now that my mother is sick, I must be at home all day and do the shopping. I have no time for myself”*  (I4) Peers sometimes cut off contact with the young person when they notice that they are now spending more time at home than usual with their ill parent. [U] *"It became like an obsession just to stay home with them [the parents] doing nothing at all. My friends stopped calling me because I didn’t call them. I simply stayed at home all the time.”*  (B2) Young people begin to experience a pressure to take care of domestic responsibilities in the house when their parent has cancer. [U] *"Just being around the house helping out . . . there was so much to do around the house that I just stayed around the house."*  (C5) Young people experience their parent's temperament and parenting change. This can include limiting the young person's socialisation. [U] *"My mom was meaner.. was more strict... and did not want me to go out."*  (D2) Some young people choose to stay home to spend time with their ill parent to process the fact their parent will die. [4] *“I chose to be at home during the days—There was a lot to cope with then, that she wouldn’t survive"*  (G3) Young people's socialisation can be limited by caring duties such as babysitting or cleaning. There is an acknowledgement by the young person that they essentially become a 'carer' even if they are not officially categorised as one. [U] ‘*I have to do much more . . . erm . . . I don’t really go out as much . . . look after my little brothers sometimes . . . do more cleaning . . . that would be me [a carer] but I won’t class myself as one"*  (G1) There is a sense of anxiety that if young people do not stay close to home that they may not be able to assist their parent if they need support. [C] *"if I’d go too far, I wouldn’t hear him call if he needed something"*  (H6) Some young people might prefer to avoid home life when their parent has cancer and instead spend time friends and go to parties. However, some young people believe that they would end up regretting this choice over spending time at home with the ill parent. [C] *"They don’t want to be at home with their parents cause they don’t want to see their parents sick, so they go out to parties and that. But later on, they regret it because they know that it was the wrong thing to do."*  (J1) Young people may take on driving responsibilities when their parent is ill. [U] *“When I got my permit two years ago, I would drive my mom and sisters everywhere. They would need to go, because she [mom] wasn’t able to drive”*  (J5) Socialising is limited by practical issues such as parents needing the car for doctors’ appointments. The young person feels stranded at home and may miss out their parent supporting them at sports games. [C] *“I don’t have a car, so like if [mom’s] out somewhere with dad at the doctor’s and I can’t— I can’t go any-where stranded at home, and it can be tough when you know parents were gone. I mean (...) stuff like sports, your parents always, can’t always come.”*  (J6) Young people must sacrifice time with peers to support their ill parent. [U] *"If I wanted to go over to see friends, but my mom wasn’t feeling well, I had to stay here with her. I couldn’t go, and all my friends out doing stuff, and I had to stay here with my mom. "*  (J7) Young people assume that the reason their socialisation is limited by their parent is due to a fear of infection while they are immunocompromised. (U) *"I don’t know why the real reason is, but she thinks that I’ll probably get sick, and I couldn’t go to any one of those things [activities with friends], because I could get sick, and my mom can’t—couldn’t like risk getting sick, too, with like chemotherapy."*  (J12) There is a pressure to become a second parent at home to support the well parent. [U] *"I was kind of the second parent, the second mom. My dad was there still but— So taking care of everyone and just making sure the house is running okay, with help on laundry, help with anything they really needed to be done, feed the dogs, or just the small things [so that they could]de-stress [mom]."*  (M6) Parental cancer can cause a role reversal between the ill parent and the child. Suddenly the young person is caring for their parent and providing emotional support to them. [U] *"she was at her very vulnerable stage I think it felt like the mother child relationships had reversed (...) I felt like I was the one kind of caring for her, I was the one making her feel better, she was the one crying onto my shoulder (...) she became a young child, a very vulnerable young child,scared vulnerable child and I just had to all of a sudden deal with this, make her not feel so scared,I had to comfort her at times I wasn’t sure that she really recognized me"*  (L6) Young people feel guilty when they are not at home to be with their ill parent. It is hard to engage in rites of passages such as University 'Freshers' when a parent is ill. [U] *"I felt guilty for being away, I felt guilty [...] it affected like my whole first year at least as if as domino effect it affected my second year [....] like freshers an all that stuff we were supposed to be just having fun and meeting loads of people and getting drunk and stuff"*  (N2) After school young people experience a second shift of domestic duties. At school they are treated as a teenager but at home they are expected to be a grown up. This is overwhelming. [U] *"When I wake up, it is a school day, but when I get back from school, it is basically like a workday because I am staying home, I am watching over the house, and I am taking care of everything there.It is kind of like I am a teenager and a grown-up.I go to school as a teenager. I get back, and I am not a teenager anymore. I am a grown-up, you know, and that alone, it could overload anybody’s head just thinking about it all"*  (N4) Young people can become emotional and experience separation anxiety when away from the ill parent. Typical adolescent activities like watching a football game feel trivial when parental cancer is going on in the foreground of their life. [U] *"I started crying on the bus because I wanted to go home, and I don’t know, it is hard. I feel like I should be home with her, and instead, I have to go to a stupid football game."*  (N5) It is essential to time with the ill parent over doing other things as time is limited. [U] *"Instead of blowing off things and going to hang out with your friends, actually be happy and stay home with your mom and cuddle with her and hold her hand— Because I know she is going to be gone soon, and this is all the time I have left [crying]"*  (O4) There is a pressure on older children in the family to provide emotional support to younger siblings. [U] *"More than ever before, I had to think about how to provide support as an older sister. I realized I had to do that when I saw my younger brother crying. [. . .] In my heart, I realized I needed to take care of him well, more than ever before. "*  (Q2) It is a struggle to decide between spending time with the ill parent or doing important tasks such as homework (U) *“That was the thing, every time you sit at the table with dad [parent with cancer] and want to stay a bit longer or something, then it’s like, okay, I want to, but I also have to study. So that was like a struggle every time.”*  (Q4) Caregiving can be lonely and there is a pressure to be stoic (C): *“There’s this feeling that I should be at home and . ..It’s just … I felt like I was supposed to be alone at home and do the laundry and such. I don’t really think they know how I’m doing.”*    (Q11) There is a sense of being stuck in the house, in the atmosphere of cancer, which can drain the young person’s energy mentally and emotionally (U): *“If you’re at home day in, day out, it feels like energy is being sucked out of you. Not because of my mum or stepdad, but because you’re constantly in that ‘cancer atmosphere’. My sister goes to Dad’s house, [parents live apart] to recharge her batteries before coming back."* | *1.2 Caring responsibilities limit social life.* |  |
| (C1) There is an assumption among young people that it is not appropriate to talk to friends about parental cancer and most people would not share this information. [U] "*Most people don't really talk about situations like this you can't go to your friends and say, hey my mom got cancer."*  (D5) Young people might want to avoid talking to other people about their parent's cancer. There is an instinct to push people and information about cancer away. [U] *"I didn’t want to talk about it—I just wanted to shove it away, I wasn’t exactly the most receptive person during that time"*  (G4) Some young people prefer to keep parental cancer a private matter at school and avoid speaking about it where possible. They don't see the point of friends being aware of parental cancer. [C] *‘I don’t really mention it at school. I mean a lot of my mates know about my dad. . . . . .y’know sometimes we get on the topic about. . .you know. . .family and stuff and it’s just. . .but like if I can avoid speaking about it in school I rather would. . .It’s just. . .you know. . .I don’t really like. . .I don’t see the point in my friends needing to know what’s going on in my personal life’*  (M4) Even when there are people to confide in about parental cancer, young people don't feel like talking to others in case their reaction causes more anxiety. [U] *"I had people that I know I could talk to about it but I didn’t really want to talk to anyone about it because I felt like their reaction would make me feel worst like I felt like they would scare me more"*  (M5) Young people do not appreciate pity from their peers. [U] *“That was my one big thing that I didn’t want like people saying ‘oh you poor thing like going home cleaning, going home cooking'"*  (M13) They fear their peer’s reaction to parental cancer. Young people do not want sympathy or their peers to be too cautious about what they should say. [U] *"I just thought they would have expected it to be just really bad (...) I didn’t want to see their reaction when I talked to them about it and I didn’t want any sympathy from them (...) I don’t want people always watching what they say or feeling guilty if they let some sort of comment about it and wondering how that would affect me..."*  (L2) Young people isolate themselves from peer support even when friends are attempting to reach out to them. [U] *"I'd made really good friends last year they [...] would come up to my room and knock on the door and [...] I just stayed in bed and pretended I wasn't in"*  (N1) They may not want peers to ask about their parent's cancer to escape the reality of their prognosis. [U] *"I don't want to deal with hearing about my mom and people go, “How is your mom doing?” (...) Then I just tell them I don’t want to talk about it because I feel like I shouldn’t be telling them news that we are not for sure about until she is actually gone and we can still hope for the better"*  (P8) Young people can worry about upsetting others by discussing parental cancer in case they are experiencing the same issue. [U] *"I am grand cause my mother is fine but like I don't want to tell people now cause like I am just maybe feel it like they might be going through the same situation, or they have lost a family member or something [...] so I kind of watch what I say to people"*  (P3) There is a sense that talking about parental cancer makes it real. [U] *"I didn't want to talk about for so long because once I said it out loud it was really, it was real and I just didn't want that to be the case at all …"*  (P4) It is more challenging to discuss parental cancer with friends in person. It is more easily facilitated by technology. [U] *"I told my best friend, one of my best friends. I went to her house and we were just chatting as normal and then I knew I had to tell her, like this was at the very beginning of Mum's diagnosis but I just wasn't able to say it, I just didn't want to say it. So actually, when I got home I just sent her a really long text and I just really wasn't able to say it in person"*  (P2) It takes time to feel comfortable enough to tell friends about parental cancer. [C] *"I thought about telling people and....I came close to a few times, but I didn't say anything. I really didn't want to talk about it I suppose...I told my friends when I went back to school that was like, three months later […] I don't think I told anyone but my friends but, as I said, that was a few months later"* | *1.3 Not opening-up and rejection of peer support.* |  |
| (A1) For some adolescents there is a desire to be 'lonely' or spend time retreating into their own world. (U) *“I am in my own world, I don’t want to speak with anyone, and I want to be lonely."*  (H5) It is necessary to have some 'me time' to actually process the reality of parental cancer. [U] *"I had time to myself and all that so I could think about what was happening around me and I kind of did this ‘me time’ so I could get used to what was happening."*  (M12) It is necessary to withdraw from others to deal with difficult emotions and bad days. [U] *"I was upset all through, but I wouldn’t be constantly upset I would just have bad days emotionally(...) I would normally just be upset just for a few hours (...)I’d go into my room really. I wouldn’t hang around; I would go away”.*  (M1) They experience a loss of identity to parental cancer and do not feel like themselves. Prior to this experience they were excited to go to parties but now they just want to be alone. [C] *"Like before I was always up for going out or if there is anything like a party or going out for the night or something, I would be always first in line straight away oh yeah definitely. After sometimes I just wanted to be by myself for a bit (...) there were several times during first year that I just sat there in my room. I remember a period of like three days where I just didn’t leave my room. Just cried a lot. Just stuff like that, I wasn’t myself socially or personality wise”.*  (M14) There is a strong desire for solitude when experiencing parental cancer. [U] *“I just wanted to be left alone "*  (N6) Young people may disassociate from the people around them by tuning out and listening to music. [U] *"[music] gives me that comfort and makes you like in your own world"*  (Q9) It is necessary to take time alone to process feeling sadness (C): *“Sometimes I just feel like being sad, without … yeah … Like I just don’t feel like being happy. Then I just want to be in my room, and just let it be.”* | *1.4 The importance of alone time.* |  |

**Table 2: Synthesized finding 2.*Young people experiencing parental cancer are lonely and face overwhelming negative emotions alone.***

| **Findings (n=17)** | **Categories (n=2)** | **Synthesised Finding** |
| --- | --- | --- |
| (A3) Young people experience an impending sense of aloneness when they think about the prospect of their parent dying. [U] *“I think if my father dies, I’ll have no-body else.”*  (F3) A feeling of loneliness despite the presence of others. [U]. *"Everyone was there at the parting at the hospital, but we were all alone in a way."*  (P1) There is a burden of feeling alone when navigating parental cancer. [U] *"Sometimes I’ll just feel alone, like I’m the only one dealing with this and it’s like having the weight of the world on my shoulders"*  (K6) It is worrying to deal with the uncertainty about who would take care of the young person if their parent died or got more ill [U] *"I worry about what would happen to me if she died? Or what would happen if she had a bad recurrence. In some ways that one’s almost even worse because I know what would happen. She’d have tests .... But then I don’t know what would happen if she was hospitalized or something."*  (G2) There is a feeling of powerlessness against parental cancer. It is like a wave that cannot be stopped and an awareness of this building. [U] *"It’s like a wave. . .it’s like some kind of wave where it just keeps building and keeps building. You know something’s gonna happen and then it suddenly does and it’s like, it washes over you. . .I don’t think about it and then something finally hits and I get shocked by it. . .and it’s not nice but there’s not much that you can do against it’"*  (H4) It is overwhelming to consider the different outcomes parental cancer might lead to and challenging to sit with this uncertainty. [C] *"It would build up because...there’s one thing that will happen and there’s a billion things that won’t happen and you don’t know which is which. So there’s a hundred million different scenarios that you’ve got to consider, which is overwhelming when you are 15.”*  (K8) There are feelings of hopelessness and a realisation that parental cancer is beyond the young person's control. They cannot intervene or change the situation or improve it. [U] *“I can’t seem to change ... well, I can’t make it (the cancer) end. It’s sort of beyond my control. But I can’t make it better, so it’s one of those things”*  *"*  (K5) Fear makes it hard to talk about cancer with other people. [U] *"I guess my own fears, death really ... stops me from talking about it"*  (Q15) Young people feel there is no obvious professional help for them which results in feeling alone (U): *“I have missed knowing where I can get help. If there was a brochure, that would already have been something. If you don’t know what there is, you can’t access it. My dad [parent with cancer] has a psychologist, but I don’t really feel like there’s anyone I can go to.”*  (Q16) Some young people find out about the diagnosis when they are alone (U): *“And then she [parent with cancer] just it was probably malignant. So yeah, at that moment, I was alone in my dorm, which was not ideal. I might have just preferred that my parents had told me face-to-face.”*  (Q18) COVID-19 intensified feeling isolated when experiencing parental cancer (U): *“Covid made it extra hard for mum, but also for me. If she’s in hospital, you can normally still visit. But during covid, sometimes I couldn’t see her for eight weeks. You can video call, but still, it is not the same.”* | *2.1 Dealing with feelings of hopelessness and fear alone.* | Young people dealing with parental cancer often feel lonely and described struggling to cope with negative emotions alone. There is a strong sense of feeling unrecognised, and powerless against their current set of circumstances. |
| (A4) There is a feeling of being overlooked at home. No one checks in on the young person to see if their needs are being met because the cancer diagnosis changes family life. [U] *“Nobody asks me what I have been doing? Nobody asks what do you eat? Or what do you wear? Or where have you been? Before the disease of my father when I was late, everybody asked me about it, but now I am ignored at home. Cancer changes the family”*  (M8) They might decline the opportunity to talk to a professional counsellor due to feeling unworthy of help. [U] *"there was help for me at the time but like my mum gave me the name of a counsellor but I had known about that before and I didn’t use it because I didn’t feel like I deserved it"*  (O5) There is a sense that there is nobody to confide in even when the young person wants to talk about parental cancer and their feelings. [C] *"Um, like, I felt in my heart that, I know, I should try not to inconvenience the people around me, but, even so, somewhere, somehow, I know, even though they were not someone I could talk to about that, I still wanted to.* (C2) Young people experience feeling unrecognised for unseen work and support they provide for their family. Society only recognises the diagnosed individual as a 'survivor’, but cancer impacts young people too when their parent is ill. [U] *"I don't really think we get recognition. I think they get recognition for being survivors which they should, but we really don't because we are behind the scenes. But we really do a lot also. Like one day I was upset because I felt like no one was giving me recognition for what I was doing."*  (E6) It is difficult to experience teachers overlooking young people at school rather than acknowledging that they are feeling upset about their parent's cancer. (U) *‘‘I would have liked them to sort of, I don’t know um acknowledge that I might actually be upset [rather than] ignore it which is what I feel they are doing’’*  (G5) Young people urge healthcare professionals to realise that it is important to speak to the whole family including children about parental cancer. It would be best to do this using lay language. [U] *‘speak with family and children because obviously we’re children and they’re like ‘oh yeah you might not understand as much’ but take that time to sit down and tell them and explain what’s happening, obviously in like child words but just take the time to acknowledge that there’s kids in the family as well and it’s not just all about the fancy words and the adults’*  (H3) There is a feeling that it is selfish to share difficult emotions with the ill parent unprompted. It would be helpful if the parent asked how the young is feeling so they do not feel guilty for bringing themselves up in conversation. [U] *"When she talks to me about her problems it makes me feel selfish if I say well, I am feeling bad or just change the subject to me. So sometimes I just wish she would ask me, so I didn’t have to feel like that in a way and have to change the conversation to me"*  (I2) There is a pressure to hide negative emotion at home such as feelings of depression. Young people feel their parents do not need to witness this. [U] *"I don’t act like I feel low and depressed when I’m at home, because I think that’s not what they need."*  (Q1) There is a pressure to pretend to be coping well in front of parents and family. (U) “I did it on my own strength. Well, I could talk about it at home, but yeah, I just did it less. Yes, to keep myself strong in front of my mom [parent who has cancer], so that I didn’t show that things were also difficult for me.”  (M3) Young people invalidate their own feelings - they feel upset about parental cancer but simultaneously guilty for having these emotions. They question if they are allowed to 'feel bad' or not [U]. *"I usually called my mum but obviously she is going through enough...cause you feel like, it’s a combination of feeling crap because it’s such a bad thing and then you feel guilty cause it’s not really happening to you, so like “okay I feel bad but should I feel this bad, how bad am I allowed to feel?”"*  (O6) Some peers encourage friends experiencing parental cancer to hide their emotion from parents [U] *"I was noticed by two people (friend and boyfriend). They were people who seemed okay to talk, so I said (about my mother’s illness), but they said you should not cry in front of my mom I did not cry so much in front of my parents (because my boyfriend and a friend told this)"*  (Q6) Teachers not understanding parental cancer is upsetting (C): *"Like when dad was admitted two weeks ago, I had a test the day after and hadn’t really been able to study. So that is hard to combine sometimes. And one teacher was understanding and allowed me to sit the test another time. But another teacher wasn’t understanding at all and made me do the test immediately.”* | *2.2 Feeling unrecognised and unworthy of help.* |  |

**Table 3: Synthesised Finding 3: *Encountering a lack of support and communication at home.***

| **Findings (n=23)** | **Categories (n=2)** | **Synthesised Finding** |
| --- | --- | --- |
| (E3) Young people feel as though they can no longer speak freely with the ill parent and they have to think before they speak to avoid upset. This is compared to when an item is breakable and you must be careful with it. [U]  *‘‘Like um you know.it’s like you know when something can break easily so you’re really careful with it? It was like that and like in the beginning it was hard that you felt that you had to think before you spoke a lot about what you were gonna say just in case it upset her.’’*  (E5) There is an avoidance of family communication about cancer even when communication about other things is good. [U] *‘‘We’ll talk about most things but when it comes to dad’s sort of thing it’s a bit more of a delicate subject’’*  (E1) There is a pressure at home for young people to keep their feelings about cancer to themselves. [U]*‘‘The whole idea is to keep quiet about it’’*  (F2) Young people may struggle with ruminating about cancer in the absence of communication [C ] *"It was the only thing you thought about, but yet we were not talking about it, everyone just went around and was...very tense."*  (H7) There is a concern that if young people are left out of the information loop about parental cancer they may feel isolated and become suicidal [U] *"(Parents), try not to disconnect yourself. If you go too long without telling the kids what’s going on, they’ll start to worry and have stupid thoughts, they think about suicide and stuff. I know a couple of friends that I’ve had to talk out of suicide and it’s because their parents weren’t telling them what was going on, they felt isolated”.*  (I3) Young people try to guess why their parent may not communicate about cancer. It could be because they don't want to think about it and are worried about dying and leaving their child behind. [C] “*Maybe she didn’t want to think about it, that she was about to die. Maybe she was anxious about dying and didn’t want to leave her son."*  (J3) It is challenging to ask a parent for information or updates about the cancer as they can be dismissive. Young people feel it is harder to not know what is going on and would rather not be shielded from this reality. [U] *"It’s hard to ask her because it seems like she just dismisses me really quickly, because she doesn’t want it to hurt me, but it’s just like the wondering about it feels like it hurts me more than just knowing what’s actually going on."*  (K7) Young people have an awareness that the ill parent may be experiencing a burn-out when discussing the cancer. This leads to less communication with the young person. (U) *"Well, I know mom is already, she’s tired of (talking about cancer). She doesn’t really want to talk about it a lot probably, because she talks to all of her doctors, and she talks to her parents about it. So, I don’t feel like I need to talk to her a lot about it."*  (M9) They may choose to not communicate with ill parent about cancer to avoid scaring them [U] *“you couldn’t talk to her because she was so sensitive about it and everyone was really very scared to talk about it"*  (O1) There is a desire to completely shield oneself from cancer and avoid thinking or talking about it. [U] *"I did not even talk about it with my family. Well, I do not want to think about it."*  (02) Young people may have to deal with the life transition from school to university on their own when a parent has cancer. There is a worry that others may think the young person uses parental cancer as an excuse. [C] *"She [my mother] did not know what I was hiding, that I really did not feel like going to T. University. I finally told her later. It was like, I was enduring it alone, and I could never say it. I was always, as if I say it, they will probably think that I am using this [my mother’s cancer] as a reason to run away. "*  (P1) The young person avoids discussing personal problems with their ill parent. They would rather discuss it with people outside of the family. [C] "*No probably I wouldn't like to talk to her too much about problems that I have. I like to keep that to myself really or talk to friends rather than to Mum […] my boyfriend and then my friends, they would be the main ones"*  *(Q8) Young people may start feeling infantilized by parents not sharing information about cancer (U): “I’d rather they’d just tell me medical results straight away than keep them from me. I’m not a child anymore."* | *3.1 Constrained family communication* | Young people feel a pressure to cope with parental cancer alone even when around family. Parents can become unavailable; siblings may cope differently and there is a dearth of communication about cancer. These circumstances cause young people to feel isolated and in the dark about cancer. |
| (B1) When a parent has cancer there is a loss of an important figure of support. It is no longer an option to cope with emotions by reaching out to the ill parent. [U] *"I had to work it out somehow myself, and I couldn’t call my Mom to talk to her (like I normally would) because I’ll get emotional."*    (D1) Once young people receive information about their parent's prognosis and diagnosis they can begin to count their parent out as a source of support for them. Grief begins before the parent actually passes away. (U) *"After receiving information about the illness it was like Mum died. I counted her out as being there for me"*  (D3) Within a family, siblings can respond to cancer differently and not support one another through the experience.(C) *"I didn’t talk to my brother about it at all—I knew that I wouldn’t get an answer—he thought it was hard, he shut himself out, it just hadn’t happened."*  (F10) When one parent becomes ill, the other is unavailable too as they must support their ill spouse. [U] *“One parent was always helping the other parent out with whatever was going on.”*  (J4) Parental cancer can cause a distant relationship between the young person and the ill parent due to the parent changing. They may become confused and unable to have a serious conversation anymore leading to a changed relationship with their child. [U] *“I mean, we’re pretty distant now because [dad’s]not the same person, obviously. He’s really confused, and you know, it’s like, you can’t really have a serious conversation with him because he can’t always grasp it, so it’s kind of tough”*  (J8) Parents can have a lack of time for their children when navigating cancer and work life. This can lead to the young person feeling they don't get enough time with their parent to talk about their day to day life. [U] *"Mom works full time, and she also has to deal with cancer, so she can’t do a lot of stuff, so that kind of,sometimes it can turn into like arguments, and sometimes you don’t have enough time with her to just like talk about like how her days were, like how our life is, and that can be hard sometimes.You lose, like touch."*  (J9) Young people may feel misunderstood by their parent [U] *"[Mom] not understanding jokes at all or sarcasm, so when I try to be sarcastic and she thinks it’s mean and she gets all mad, but that kind of stuff, small things."*  (Q14) It is difficult to spend time together as a family now, as there is a sense of not being able to see the parent as visiting hours can conflict with school (C): *“If we go directly after school, we’re stuck in traffic, because then you’re at the wrong place at the wrong time. A later time also doesn’t work, because visiting hours are until eight. If the visiting hours would be extended a little longer, it would help to be able to be with dad.”*  (L7) There is a feeling that there is no one to turn to at home for support [U] *"I’ve never spoken to anyone about it properly apart from my mum but I wouldn’t tell her half of this so like (yeah )I didn’t want to worry her more and I don’t talk to my sister much yeah and my dad is nothing like an emotionally open person so I wouldn’t really haven’t talk this much with anyone."*    (L2) Young people decide against opening up about challenging life transitions such as going to university with their parent. [U] *"I was going through a lot when I first went into University, I don't think I was prepared for it at all and I didn't talk to my mum about that anymore, like I really just tried to help her."* | *3.2 Changed family dynamics and support.* |  |

**Table 4: Synthesised Finding 4: *Communication and social support protect young people.***

| **Findings (n=23)** | **Categories (n=2)** | **Synthesised Finding** |
| --- | --- | --- |
| (H2) Some young people feel less affected by parental cancer compared to others as their healthy parent provided such strong support and checked in on them. [U] “*I don’t think I was affected as much as other people have been by it cause....Dad was always there to support everyone....When Mum went into hospital treatments, Dad was always there to talk to me and my brothers to see if we were going well."*  (I1) When family communication is strong, support from elsewhere is not needed [U] *"We didn’t need to go somewhere else to talk, because we have always had each other, and we can talk about anything."*  (K2) It helps to talk to the ill parent about cancer. [U] *"Like other people’s parents, I know that they’ll be having their parents be having cancer and they don’t really talk to their moms about it and stuff. I talk, I talk to her about (cancer), like talk to her—talk to her ... and it helps"*  (L4) It is helpful when the young person is treated like a grown up in the situation. [U] *"“[...]my father would have treated me with a lot of respect,he used to tell me that he was very proud of me[...] he was treating me like I could comprehend as if I was an adult[...]"*  (L3) Cancer can increase family communication and closeness [U] *"Its opened things up to talk about emotions more, its opened things up being more sentimental and it has made us closer, very much so."*  (P6) When informational needs are met young people feel less anxious [U] *"I'd rather that they tell me everything than they hold back because I would be more anxious and more worried if they held back. I felt that I was better able to cope and deal with it if I knew everything that was going on because I could have the bigger picture"*  (Q5) Young people can be protected by sharing honest information with them (U): *“Dad [parent with cancer] is a straightforward and honest person. It would be weird if he didn’t share things immediately. If my family wasn’t open, with close bonds, I wouldn’t be doing so well.”*  *(Q10) Having a sibling to confide is very helpful (U): “I have a sister and that’s an advantage. She’s my best friend. She’s in the exact same situation as me and also, she’s only a year older than me. So we understand each other completely. I can really tell her everything. And she can tell me everything as well.”*  (Q17) Young people feel strongly that no information should be withheld from them (U): “*I think it’s important that you just know everything and nothing is kept from you. Yes. That’s what I consider most important. Because if things are being kept from you, and you don’t know something, and then something happens that you don’t expect and that’s not nice at all.”*  (Q20) It is important to talk openly with family without any fear or embarrassment (U*): “My stepdad and I can talk about everything without any embarrassment or fear, about the whole situation with my mom"* | 4.1 *Communication at home is important.* | Strong family communication, help from teachers or counsellors, and being open to friendship support—especially from peers who have experienced parental cancer first-hand—are some of the factors that may shield young people from feeling lonely when their parent has cancer. |
| (O3) When young people have the opportunity to speak to a grown up outside of their family they feel relieved to be confiding in someone and appreciate being noticed. This relieves feelings of aloneness. [U] *"There was also one time before a big club meeting. I guess since it was my advisor, it somehow seemed like they knew what I was going through, and they listened to what I had to say. [. . .] Um, like, they would notice I had something I could not say to others and they would talk to me. It was as if I was not alone."*  (P7) Professional support is important to young people. It was useful to be able to talk to someone outside of family life who was objective. [U] *"I did crash when Mum started to go to chemo and I had to go to therapy and ...but I think it was really the best thing for me because I just cried and talked about everything. I think it really did help me being able to talk to someone that was really objective to the situation"*  (P5) It is helpful for teachers and school staff to chat to the young person about parental cancer [U] *"My religion teacher and the school chaplain like I'd have chats every week, like she'd spot me in the corridor and she would be like ‘(name), would you like to come for a chat?’ [...] they were great support"*  (J2) Communication about cancer can provide relief from rumination. This helps to alleviate stress. [U] *“I think it [talking] like re-leases the stress or like the thoughts that are in my head.”*  (M11) In retrospect communication and support from other people would have been helpful (U) *"“I didn’t like to make it seem like it was such a big deal, like I really needed help but (...) it probably would have been better if I had just talked about it maybe once or twice”.*  (O7) Some young people would like to directly speak to healthcare professionals involved in their parents medical management about the prognosis and stage of the cancer. [U] *'I kind of wanted to hear what stage my mother’s condition was, or the severity, not from my mother, but from the actual doctor."* (Q7) It is helpful when medical professionals acknowledge the young person is present and is impacted too (U): *“I just like it when they [the medics] ask me how I’m doing when I’m at the hospital. For me, a sincere “how are you?“, is already a sign of acknowledgement that you’re there too.”* | *4.2 Support from adults outside of the family* |  |
| (C3) It would be helpful to get some reassurance from another young person with lived experience of parental cancer. [U] *"I just want someone to tell me it will be ok... someone who has been through the process with their parent."*  (C4) There is a desire for a peer support group to be available for young people who are experiencing parental cancer. It would be useful to build relationships with other young people with insight. [U] *"Maybe if you went to the group with the same people and then you build relationships... We would come."*  (E2) Young people can get support from friends who have also gone through parental illness. [U] *‘‘I’ve been supported by friends who have been through similar experiences’’*  (E4) Friendship is important to young people and a friend can get supporting the young person 'just right’. [U] “*a friend can get it just right’’*  (Q3) It means a lot when a friend asks how the ill parent is doing (U) “*It [parental cancer] doesn’t always have to be the topic, but it’s nice if my friends ask how my mum is doing after three weeks or at an unexpected moment. I like that they ask about it … their closeness, their concern.”*  (Q12) For some young people, their friends noticed something is wrong and gave comfort to the young person when they heard of the diagnosis (U): “*The next day, I didn’t go to school and just stayed home a bit. But in the evening, I did go to athletics to chat with my friends there about it … I started to cry and they asked what was wrong, and I said that my dad had lymph node cancer, and they all hugged me, which was comforting.”*  (H1) When a friend knows the right thing to say it is very comforting for the young person. [C] *"I found that my friends know me like, really socially so they know, what to say"*  (H8) It is helpful to meet other young people experiencing parental cancer. This is a reminder to the young person that there is support around them and they can also provide to support to others in a similar position. [C] *"I just like meeting new people and helping people through what pretty much I’m going through at the same time, and knowing that other people out there are going through exactly, well almost exactly, what I’m going through. And there is support out there for anyone like me if I need it"*  (J13) Friends with shared experiences of parental cancer can become a source of information for the young person. (U) *“I have many friends whose parents also have had cancer, and then we share information on many things— (...) It’s like, it’s totally different because we can relate."*  (K3) Friends can cheer up and reassure young people experiencing parental cancer by letting them know that they are a source of support for them. [U] *“My friends just cheer me up, like they’ll play with me and say, like, “don’t think about (cancer) that much.” They’ll talk to me about (cancer), and they’ll just say, “it’s going to be okay and we’ll be there for you when ... he (dad) is gone.”*  (M7) Friendship might protect young people against turning to maladaptive coping mechanisms such as drugs and alcohol when dealing with parental cancer. [U] *"I think maybe provide more services for adolescents like I was lucky, I had a lot of support in the school with my friends but there could be some teenagers out there that they just don’t know where to turn to and then they could turn to things such as alcohol or drugs, that’s not right like"*  (L5) Roommates can provide good support to the young person [U]. *"when my mum went through chemo I had a lot of support through like my roommates and stuff, they were very good with it"*  (N3) Friendship is an important source of support at school when the young person becomes overwhelmed with emotion [U] *“I was in the middle of class, and I started crying, and, honestly she [a friend] just stood up and gave me a hug, and it really did, it made everything better.”*  (P9) It is helpful when friends understand the young person's priorities shifting toward domestic tasks when a parent has cancer. [U] *"they would just understand that and let me talk and they'd let me do things I needed to do to cope like clean really extensively the house top to toe every day, cook for them, like I like to do things like the day my Mum got diagnosed with cancer [...] I just had to do things to get my mind off things. They'd allow that to happen"* | *4.3 The importance of friendship and shared experience* |  |
